# Next Generation Sequencing Assay for Detection of Circulating HPV DNA (cHPV-DNA) in Patients Undergoing Radical (Chemo)Radiotherapy in Anal Squamous Cell Carcinoma (ASCC)

**DOI:** 10.3389/fonc.2020.00505

**Published:** 2020-04-17

**Authors:** Jen Y. Lee, Rosalind J. Cutts, Ingrid White, Yolanda Augustin, Isaac Garcia-Murillas, Kerry Fenwick, Nik Matthews, Nicholas C. Turner, Kevin Harrington, Duncan C. Gilbert, Shreerang Bhide

**Affiliations:** ^1^Radiotherapy and Imaging, The Institute of Cancer Research, London, United Kingdom; ^2^Department of Clinical Oncology, The Royal Marsden Hospital, London, United Kingdom; ^3^Department of Clinical Oncology, St. Georges Hospital NHS Trust, London, United Kingdom; ^4^Department of Clinical Oncology, Brighton and Sussex University Hospitals NHS Trust, Brighton, United Kingdom; ^5^MRC Clinical Trials Unit at UCL, London, United Kingdom

**Keywords:** Plasma HPV-DNA, chemo-radiation, anal cancer, locally advanced, response prediction

## Abstract

**Background:** Following chemo-radiotherapy (CRT) for human papilloma virus positive (HPV+) anal squamous cell carcinoma (ASCC), detection of residual/recurrent disease is challenging. Patients frequently undergo unnecessary repeated biopsies for abnormal MRI/clinical findings. In a pilot study we assessed the role of circulating HPV-DNA in identifying “true” residual disease.

**Methods:** We prospectively collected plasma samples at baseline (*n* = 21) and 12 weeks post-CRT (*n* = 17). Circulating HPV-DNA (cHPV DNA) was measured using a novel next generation sequencing (NGS) assay, panHPV-detect, comprising of two primer pools covering distinct regions of eight high-risk HPV genomes (16, 18, 31, 33, 35, 45, 52, and 58) to detect circulating HPV-DNA (cHPV DNA). cHPV-DNA levels post-CRT were correlated to disease response.

**Results:** In pre-CRT samples, panHPV-detect demonstrated 100% sensitivity and specificity for HPV associated ASCC. PanHPV-detect was able to demonstrate cHPV-DNA in 100% (9/9) patients with T1/T2N0 cancers. cHPV-DNA was detectable 12 weeks post CRT in just 2/17 patients, both of whom relapsed. 1/16 patients who had a clinical complete response (CR) at 3 months post-CRT but relapsed at 9 months and 1/1 patient with a partial response (PR). PanHPV-detect demonstrated 100% sensitivity and specificity in predicting response to CRT.

**Conclusion:** We demonstrate that panHPV-detect, an NSG assay is a highly sensitive and specific test for the identification of cHPV-DNA in plasma at diagnosis. cHPV-DNA post-treatment may predict clinical response to CRT.

## Introduction

Radical chemoradiotherapy (CRT) is a standard of care for patients with locally advanced anal squamous cell carcinoma (LA-ASCC) (1). The majority of patients with loco-regional disease will be cured, but patients with residual or recurrent disease may be salvaged with radical surgery making on going assessment of disease imperative. Following CRT there is no standard recommended single modality for response assessment. ESMO-ESSO-ESTRO guidelines recommend a combination of digital rectal examination (DRE), MRI of the pelvis for loco-regional response and CT/PET-CT for systemic response (1). LA-ASCC are slow to regress and 26 weeks is said to be the optimal time-point for response assessment based on data from ACT II study (2). Positive predictive value of these modalities is sub-optimal and can lead to unnecessary biopsies in some patients. Delayed histological confirmation of residual disease in others may result in salvage surgery with R1/R2 resection (3, 4), which has a direct impact on outcomes. Furthermore, uncertainties surrounding presence of residual disease up to 6 months after CRT result in significant patient anxiety. Therefore, better predictors of “true” residual disease are required (5).

The majority of LA-ASCC's are causally related to the human papilloma virus (HPV) (6) which confers prognostic benefit over the few HPV negative cases (7). In the tumor, HPV DNA is integrated into the host genome or is present in episomal form (8) and can be detected in the blood. Circulating tumor DNA has gained much attention as a method of investigating and monitoring tumor biology and clinical status (9, 10); cHPV-DNA can potentially be used as a detection marker for HPV-related LA-ASCC. The utility of cHPV-DNA in monitoring disease response following radical chemo-radiotherapy in LA-ASCC has not been extensively evaluated. In a single prospective study, Cabel et al. measured circulating tumor DNA in pre and post-CRT plasma samples in 33 patients with LA-ASCC using a droplet digital PCR (ddPCR) based assay with 88% sensitivity for detecting HPV DNA at baseline (11).

We developed an ultra-sensitive HPV DNA next generation sequencing (NGS) assay, panHPV-detect, with the ability to comprehensively detect circulating DNA of high-risk HPV genomes (16, 18, 31, 33, 35, 45, 52, and 58) and assess its relationship with disease status and response. We then validated the assay in prospectively collected plasma DNA at serial time points in patients with ASCC treated with primary CRT.

## Materials and Methods

Informed consent was obtained from all eligible patients with stage I-IIIB ASCC (AJCC 2007) who were due to receive radical CRT. Institutional board (Ref. no. CCR 4157) and ethics committee (Ref. no. 14/NE/1055) approved the study. Patients received mitomycin C 12mg/m^2^ d1 and capecitabine 1,650 mg/m^2^/d in 2 divided doses for 28 days (days on which RT was delivered) (12, 13). Radiotherapy was delivered using a simultaneous integrated boost (SIB)-IMRT technique as described previously (14). Gross tumor volumes (GTV) for primary disease and lymph nodes were delineated based on pre-treatment ^18^F-FDG PET-CT and MRI. Clinical target volume (CTV)_anal_ included primary tumor with a margin, CTV_nodes_ involved lymph nodes with margin and CTV_elec_ the elective nodal volumes as per the UK IMRT anal cancer guidelines. Planning target volumes (PTV_anal_, PTV_nodes_, PTV_elective_, respectively), were created from CTV's as per the UK IMRT anal cancer guidelines. Radiotherapy was administered in 28 fractions over 38 days. PTV_anal_ received 53.2 Gy (50.4 Gy for stage II disease), PTV_nodes_ 50.4 Gy, and PTV_elective_ 40 Gy.

At 12 weeks following treatment, response was assessed by clinical examination and MRI. ^18^F-FDG PET-CT was performed as recommended at the multidisciplinary team (MDT) meeting. Persistent residual disease at 26 weeks on DRE and MRI was confirmed with a biopsy.

Serial plasma samples were collected at baseline (before CRT), 6 weeks and 12 weeks following completion of treatment in Streck® tubes. Samples from one HPV negative patient, one sample of pooled plasma from a set of healthy pre-screened individuals with no cancer or history of cancer purchased from CTLS (Clinical Trial Laboratory Services), London and 19 patients with breast cancer enrolled in the prospective sample collection study (PlasmaDNA, CCR3297, REC Ref No: 10/H0805/50) were used as negative controls. None of the negative controls were known to have pre-cancerous lesions. Written informed consent was obtained from all participants.

### Blood Samples: Plasma Processing and DNA Extraction

Twenty milliliter of blood was centrifuged at 1,600 g for 10 min within 48 h of collection. The resulting plasma was then centrifuged again at 1,600 g for 10 min, aliquoted and frozen at ^−^80°C. DNA was extracted from 5 mL of plasma using the QIAamp Circulating Nucleic Acid Kit (Qiagen) according manufacturer's instructions. DNA was eluted in 50 μL of AVE buffer and stored at ^−^20°C. Plasma DNA was quantified using a Bio-Rad QX200 ddPCR system, using ribonuclease P (RNase P) as a reference gene as previously described (15).

### Tumor Biopsies: HPV16 Detection in Tumor

Formalin fixed paraffin embedded tumor blocks of the diagnostic biopsy samples were obtained. Eight 10 μm nuclear fast red stained slides and two haematoxylin and eosin (H&E) stained slides were obtained from representative FFPE blocks. Tumor content, cellularity and suitable areas of tumor were marked for macro-dissection. RNA was extracted using the AllPrep DNA/RNA FFPE Kit (Qiagen) followed by cDNA synthesis using the Omniscript Reverse Transcription kit (Qiagen). Evidence of HPV 16 status integration was assessed by detection of E7 expression using methods and primers described previously (16) in a 7,500 Sequence detection system (Applied Biosystems/Thermo Fisher). Any specimen producing a dCt <13 was consider positive for HPV integration.

### cHPV-DNA Sequencing in Plasma and Tissue (panHPV-Detect Assay Design)

Pan HPV-detect was designed as follows. Representative sub-lineages (17) from each of the 8 high risk HPV genotypes were aligned using CLUSTAL O (1.2.1) multiple sequence alignment programme (https://www.ebi.ac.uk/). Diagnostic single nucleotide polymorphisms (SNP) were identified for each sub-lineage (18–20) and used as a guide for primer design using the Ampliseq Designer (ThermoFisher Scientific). Eight primer sets targeting the human genes ACTINB, GAPDH, HPRT were also included in the panel to act as positive controls for library preparation and sequencing efficiency. The panel consisted of 548 primers divided into 2 pools. Ion torrent libraries were prepared using Ion Ampliseq library preparation kit 2.0 (ThermoFisher Scientific) according to manufacturer's instructions using 5 ng of tissue DNA or 3 ng of plasma DNA per primer pool. Reads were aligned to an amalgamated reference containing scaffolds for each of the HPV genotypes as well as the reference human targeting genes using TMAP on the Ion Torrent machine. Bedtools v2.23.0 (21) was used to extract on-target reads from the aligned files with a minimum overlap of 90% with amplicons in the panel. Additionally reads with a mapping quality of <30 were removed using samtools v1.2 (22). Reads were split into those covering human and HPV amplicons and coverage of each portion and each genotype was calculated individually.

We performed an initial validation of panHPV-detect by testing its ability to correctly identify the HPV sub-type in cervical cancer tissue samples. Given the rare occurrence of non-HPV16 in Anal SCC, we obtained cervical cancer tissue previously typed for the HPV sub-type using validated assays for tissue samples. Five samples for each of the 8 high-risk HPV sub-types were obtained from the Scottish HPV archive.

### Statistical Considerations

#### Sample Size Calculation

The primary end-point for this pilot study was feasibility of detecting cHPV-DNA at baseline in patients undergoing CRT with radical intent. Our pilot study in HPV+ head and neck cancer demonstrated 100% sensitivity and 93% specificity in detecting cHPV-DNA in patients' plasma at baseline (pre-treatment) using the original NGS assay (HPV-detect) (10). To prove 85% sensitivity assuming that the true sensitivity is 99% and to have 80% power given two-sided type I error of 0.05, would require at least 19 HPV+ patients, all of them expected to be labeled as positive by NGS. Since ~85% of LA-ASCC patients are HPV+, therefore, the study required 22 patients. The secondary end-point was to assess the potential of cHPV-DNA to predict response to treatment.

### Data Analyses

Correlation analysis was used to quantify the relationship between plasma HPV DNA levels at baseline with tumor volumes. Comparisons between different HPV16 assays in different tissue types were analyzed using contingency diagrams and Fisher's exact test. All statistical analyses were calculated in GraphPad Prism version 7.

In order to classify HPV+ and HPV- samples using panHPV-detect in tissue, we set a threshold whereby a sample was classified positive if there were 10 reads present from more than 10 different HPV amplicons for each sub-type. In order to assess the threshold for the number of amplicons needed for positive panHPV-detect readout in plasma—a ROC analysis was used. In the first step, HPV status was assigned in tissue using the gold standard E7 mRNA to separate the two groups. The number of amplicons with >10 reads at baseline was inputted for each patient to find a suitable threshold for this parameter. Sorting the values in both HPV+ and negative groups and averaging adjacent values in the sorted list generated a list of thresholds. Based on the ROC analysis, a threshold of 5.5 amplicons with more than 10 reads gave the greatest sensitivity and specificity and was selected as threshold for classification of plasma as HPV DNA positive (Table 1).

**Table 1:** ROC analyses thresholds and results.

**Cut-off (no. of amplicons)**	**Sensitivity%**	**95% CI**	**Specificity%**	**95% CI**	**Likelihood ratio**
>0.5000	100.0	81.5–100.0%	70	47–87%	3.29
>1.5	100.0	81.5–100.0%	74	52–90%	3.83
>2.5	100.0	81.5–100.0%	78	56–93%	4.6
>4	100.0	81.5–100.0%	91	72–99%	11.5
**>5.5**	**100.0**	**81.5–100.0%**	**96**	**78–99.9%**	**23**
>16.5	100.0	81.5–100.0%	100.0	81.5–100.0%	
>30.5	95	74–99%	100.0	81.5–100.0%	
>37	89	65–98%	100.0	81.5–100.0%	
>50.5	84	59–96%	100.0	81.5–100.0%	
>62	78	52–93%	100.0	81.5–100.0%	
>68	72	47–90%	100.0	81.5–100.0%	
>73.5	67	41–87%	100.0	81.5–100.0%	
>74.5	61	36–83%	100.0	81.5–100.0%	
>76	50	26–74%	100.0	81.5–100.0%	
>77.5	28	10–53%	100.0	81.5–100.0%	

*CI, confidence interval. The bold values stand for the threshold identified for classifying samples into HPV +ve and -ve using the ROC analysis*.

## Results

Twenty-four patients were recruited into the study. Tumor tissue (ASCC diagnosis) and baseline blood samples were available for 21 patients (Table 2). Tumor DNA content and/or the collected plasma samples were found to be sub-optimal in four patients. Complete sample sets i.e., tumor tissue, baseline and 3 month plasma sample were available for 17 patients. Of these 6-week blood samples were available for analysis in 8 patients. Median follow-up was 16.8 months (range 4–24 months).

**Table 2:** Patient and tumor characteristics.

**Variable**		**Number of cases**
Number of patients		21
T-stage	1	3
	2	10
	3	5
	4	3
N-stage	0	11
	1	6
	2	3
	3	1
Overall stage	I + II	9
	III	12
Sex	Male	9
	Female	12
HIV status	Positive	4
	Negative	17
Smoking status	Current	7
	Ex-smoker	4

Forty cervical cancer samples typed for HPV sub-type (five of each sub-type) were obtained for tissue validation, Tumor DNA from one of these failed library preparation during NGS. Therefore, 39 samples were available for analysis. panHPV-detect was able to correctly identify the HPV sub-type in all 39 samples.

Tumor tissues from 20 patients with ASCC in this study were classified as HPV+ and one patient as HPV- by E7 mRNA analyses, consistent with previous reports (23). Correlation of the tissue HPV status with detectable cHPV-DNA following sequencing of baseline plasma samples, demonstrated that cHPV-DNA was detected in 20/20 HPV+ patients and 0/21 negative controls using panHPV-detect (Table 3). None of the 21 negative controls (including patient with tumor classified as HPV-) had cHPV-DNA in plasma at baseline. This gave a sensitivity and specificity of 100% (95% CI 83–100%) and 100%, respectively (95% CI 85–100%), for all patients and 100% sensitivity (9/9) for patients with Stage I/II disease. There was no correlation between the volumes of disease (primary and lymph nodes) and cHPV-DNA levels in plasma (*r*^2^ = 0.3, *p* = 0.13).

**Table 3:** Comparison of HPV status using E7 expression in tissue and pan HPV-detect in baseline plasma.

		**Tumor HPV status—E7 PCR**		
		**Present**	**Absent**	**Total**
cHPV-DNA panHPV-detect	Present	20	0	20
	Absent	0	21	21
	Total	20 (sensitivity 100%)	21 (specificity 100%)	41
		PPV 100%	NPV 100%	

**Figure 1:**
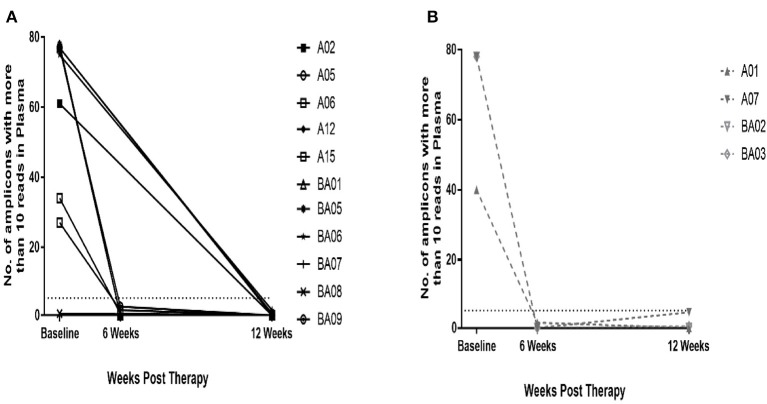
Plasma HPV-DNA levels at baseline and 12 weeks post-CRT. 14/15 patients had above threshold levels of cHPV-DNA at baseline. At 12 weeks post- CRT, cHPV-DNA levels dropped to below threshold in all 14 patients. **(A)** 11 patients had complete response on DRE/MRI and cHPV-DNA below threshold. **(B)** 4 patients with partial response on DRE/MRI at 12 weeks and below threshold cHPV-DNA; further DRE/MRI at 6/12 months showed no evidence of relapse The dashed line on the Y-axis denotes the panHPV-detect threshold at 5.5 amplicons with more than 10 reads.

**Figure 2:**
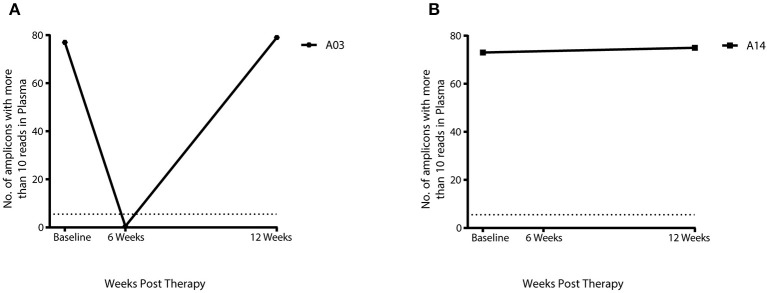
cHPV DNA levels in 2 patients with either a partial response or relapse using panHPV-detect. **(A)** In patient A03 cHPV-DNA was below threshold levels at 6 weeks, cHPV-DNA levels then increased above threshold at 12 weeks with residual disease on DRE/MRI **(B)** Above threshold levels of cHPV-DNA in patient A14 with complete response on DRE/MRI, distal relapse was detected at 9 months post-CRT The dashed line on the Y-axis denotes the panHPV-detect threshold at 5.5 amplicons with more than 10 reads.

Twelve of the 17 HPV+ patients with complete sample set had complete response and five patients had a partial response as assessed by DRE/MRI scan at 12 weeks post-CRT. cHPV-DNA levels at 12 weeks post-CRT were below the threshold of detection in 11 out of 12 patients who had a complete response (Figure 1A) and 4 out of the 5 patients with partial response (Figure 1B). Results from the 7 patients with available samples at the 6-week sample mirrored those of the corresponding 12-week sample (Figure 1B). cHPV-DNA levels were undetectable at the 6-week time-point but above the threshold of detection in one patient at the 12-week time-point (A03, Figure 2A) This patient had high index of high-volume residual disease based on MRI and clinical examination. Therefore, which was histologically confirmation was undertaken at the 12-week time-point in the interest of expediting salvage surgery. One patient, who had cHPV-DNA levels above the threshold and clinical complete response (A14, Figure 2B), relapsed distally at 9 months following treatment completion. Four patients with persistent abnormality on MRI/clinical palpation at 12 weeks and undetectable HPV DNA levels, underwent PET and EUA at 6/12 months—all were subsequently all negative for relapse (Figure 1B). These four patients and the 11 patients who had a clinical complete response and undetectable cHPV-DNA at 12 weeks remain relapse free at the last clinical follow-up (median 16.8 months).

## Discussion

We describe “panHPV-detect,” a novel ultra-sensitive method of detecting and tracking HPV-DNA from eight high-risk HPV genotypes in LA-ASCC in a study with prospective collection of biological samples. Amplicon-based sequencing of multiple regions of the viral genome enables the detection of HPV DNA at baseline with high sensitivity and specificity. Tracking cHPV-DNA in sequential samples through and after CRT predicted response and residual disease suggesting the potential of panHPV-detect to enhance clinical decision-making.

To our knowledge this is the first study to use NGS to detect cHPV-DNA in patients undergoing CRT for LA-ASCC. Cabel et. al. prospectively measured cHPV-DNA in 33 HPV+ patients and demonstrated a sensitivity of 88% (64% in stage II) at baseline (11). Cabel et al. used ddPCR for detection of a single region of the HPV genome (E7) in plasma for two high-risk HPV subtypes- 16 and 18. Our NGS assay, which is able to detect multiple HPV genomic regions across eight high-risk genotypes, demonstrates superior sensitivity of 100%. Furthermore, our assay demonstrates 100% sensitivity in 9 patients with Stage I/II disease as opposed 64% in the study by Cabel et. al. Negative controls were not used in the study by Cabel et. al. and therefore the authors did not comment on the specificity of the assay. Our study demonstrates a specificity of 100%.

Fifteen out of 17 HPV+ patients in our study had undetectable cHPV-DNA at 12 weeks post-CRT. In four of these patients who had partial response on DRE/MRI, however, no evidence of residual disease on PET-CT and biopsies at 6–12 months was observed. Therefore, cHPV-DNA levels at 12 weeks could potentially predict “true” absence of disease, avoiding the need for repeated imaging and biopsies. This would bring considerable benefit in terms of cost savings for the health service and avoid unnecessary patient anxiety.

Two patients in our study had detectable cHPV-DNA at the 12 week time-point both of whom had histologically confirmed relapse. Similarly in the study by Cabel et al. three patients (out of their 17 with post-CRT blood samples) had detectable cHPV-DNA all of whom had metastatic relapse. The ESMO-ESSO-ESTRO guidelines recommend 26 weeks as the optimal time-point for response assessment following CRT. Elevated cHPV-DNA levels at 12 weeks post-CRT in our study and the one by Cabel et al. accurately predict residual disease and/or disease relapse (5/34 patients with detectable cHPV-DNA at 12 weeks, both studies combined). Outcomes following salvage surgery for residual loco-regional disease are related to T-stage. Biopsy confirmation and salvage surgery at an earlier time-point, i.e., 12 weeks post-CRT based on cHPV-DNA levels as opposed to 26 weeks based on DRE/MRI, could potentially avoid T-stage progression in the intervening period and improve outcomes.

In patients with elevated cHPV-DNA and no evidence of local disease at 12 weeks, commencement of systemic treatment in the presence of lower disease burden, i.e., before disease becomes clinically or radiologically apparent, could potentially lead to improvement in outcomes. Identification of true high-risk patients using more sensitive assays such as plasma cHPV-DNA could aid the design of adjuvant therapy studies using novel agents including drugs targeting immune checkpoints.

This was a feasibility study to investigate the potential of a novel NGS assay to detect cHPV-DNA before radical therapy. Although it was not powered to do so, the study highlights the potential of cHPV-DNA in predicting disease response following CRT. cHPV-DNA has significant potential as a biomarker of disease response post-CRT and of disease relapse during disease surveillance for patients with LA-ASCC. All of the patients included in study had tumors causally related to HPV16. However, anal SCC can be causally related to other HPV sub-types such as 18, 33, 31, and 45 Therefore a larger study is required to test the performance of panHPV-detect in detecting other HPV sub-types in plasma. Furthermore, panHPV-detect can identify cHPV-DNA with high sensitivity and specificity enabling the use of cHPV-DNA as a biomarker to become reality, albeit further validation in larger multi-center studies is required. To this end the performance characteristics of cHPV-DNA in monitoring response will be prospectively tested in a cohort of locally-advanced ASCC as part of PLATO, the ongoing platform trial testing personalisation of radiotherapy doses funded by Cancer Research UK (24).

## Data Availability Statement

All datasets generated for this study are included in the article/supplementary material.

## Ethics Statement

The studies involving human participants were reviewed and approved by HRA-NRES-Committe-North-east-York. The patients/participants provided their written informed consent to participate in this study.

## Author Contributions

JL: samples processing, analysis, and data analysis. RC: study bioinformatician. IW and YA: patient recruitment. KF and NM: sample sequencing. NT and IG-M: input into NGS assay design. KH: study design and manuscript review. DG: patient recruitment, manuscript preparation, and review. SB: chief investigator, study design and concept, patient recruitment, data interpretation, manuscript preparation.

## Conflict of Interest

The authors declare that the research was conducted in the absence of any commercial or financial relationships that could be construed as a potential conflict of interest.
